# The Genomic Characterization of a Novel *Candidatus* Genus of the Family *Gallionellaceae,* a Novel *Candidatus* Species of the Genus *Gallionella* and Their Metabolic Potential for Iron Oxidation

**DOI:** 10.3390/ijms27198801

**Published:** 2026-10-01

**Authors:** Nikolai V. Ravin, Maria V. Gureeva, Alexey V. Beletsky, Margarita Y. Grabovich

**Affiliations:** 1 Institute of Bioengineering, Research Center of Biotechnology, Russian Academy of Sciences, Leninsky Prosp., Bld. 33-2, 119071 Moscow, Russiamortu@yandex.ru (A.V.B.); 2 Department of Biochemistry and Cell Physiology, Voronezh State University, Universitetskaya Pl., 1, 394018 Voronezh, Russia

**Keywords:** *Gallionella*, iron oxidation, MAG, Cyc2, MtoA

## Abstract

The family *Gallionellaceae* includes neutrophilic, aerobic, iron-oxidizing bacteria, many of which remain uncultured and known only from metagenomic data. In this study, two high-quality *Gallionellaceae* genomes were assembled from a ferruginous spring in the Voronezh region, Russia. Phylogenetic analysis based on 120 concatenated proteins, ANI (average nucleotide identity), and AAI (average amino acid identity) indicates that BG-057 belongs to the genus *Gallionella* and BG-364 to the genus 39-52-133. We propose the names “*Candidatus* Gallionella aquifuscii” sp. nov. for BG-057 and “*Candidatus* Dubininella ochracea” gen. nov., sp. nov. for BG-364. Both genomes encode genes for the Calvin-Benson-Bassham (CBB) cycle. Both are potentially capable of aerobic respiration due to the presence of electron transfer chain components. Both genomes encode the iron oxidase Cyc2, and BG-057 also encodes MtoA. BG-057 is potentially capable of lithotrophic growth in the presence of H_2_ and H_2_S. BG-364 is potentially capable of growth via Mn (II) oxidation; arsenite oxidation; the direct oxidation of thiosulfate; H_2_S, elemental sulfur, and sulfite oxidation. BG-364 encodes denitrification genes (*narGHI*, *nirS*, *norBC*). BG-057 possesses systems for the biosynthesis and export of siderophores, while BG-364 lacks these systems but retains Ton-dependent receptors, suggesting the use of exogenous siderophores.

## 1. Introduction

The genus *Gallionella* has a nearly two-century history of study. The first species of this genus, *Gallionella ferruginea*, was discovered by Ehrenberg in 1836 [1]. At first the species were mistaken for algae, but Winogradsky demonstrated their bacterial nature and suggested their ability to grow chemolithotrophically through the oxidation of Fe (II) [2,3]. Cells of the genus *Gallionella* are bean- or rod-shaped [4,5]. A characteristic feature of representatives of this genus is the formation of a twisted stem consisting of several fibrils covered with layers of polysaccharides encrusted with Fe (III) compounds. It is believed that the physiological role of the formation of such structures is the removal of iron oxidized during lithotrophic growth [6].

The genus *Gallionella* is currently classified within the family *Gallionellaceae*. Most members of the family *Gallionellaceae* are iron-oxidizing bacteria. They are able to use Fe (II), reduced sulfur compounds, hydrogen, and nitrite as electron donors for lithotrophic growth. Many of them are capable of lithoautotrophic growth through the Calvin-Benson-Bassham (CBB) cycle. During lithoautotrophic growth, NADH is reduced through reverse electron transport [7]. However, a mixotrophic mode of metabolism has been demonstrated in some representatives of the family *Gallionellaceae* [4]. Some representatives are able to oxidize Fe (II) under anaerobic conditions, using nitrate as the terminal electron acceptor [8].

Representatives of the family *Gallionellaceae* are difficult-to-cultivate microorganisms [9].

According to the latest release 232 of the Genome Taxonomy Database (GTDB) [10], the family includes four genera with validly described names, *Gallionella*, *Sideroxydans*, *Ferriphaselus*, and “*Candidatus* Nitrotoga”, and 14 candidate genus-level lineages. In contrast to traditional taxonomy [11,12], the genus *Gallionella* includes the genus *Ferrigenium*, and the genus *Sideroxydans* includes the genus *Sideroxyarcus* in the GTDB. Members of only three genera—*Gallionella*, *Sideroxydans*, and *Ferriphaselus*—have been isolated in pure culture [13,14,15]. However, information on most species within these three genera, and on all other genera within the family, is available only through metagenomic sequencing data. Therefore, the description of new species based on genomic data is of great importance for this family [16].

In this study, we sequenced the metagenome of a microbial mat from the Belaya Gorka ferruginous mineral spring in the Voronezh region, Russia. We then assembled the genomes of two new species from the family *Gallionellaceae* in this metagenome, analyzed their metabolic potential using genomic data, and proposed them as *Candidatus* taxa. One of the new species belongs to the genus 39-52-133; therefore, we propose the name “*Candidatus* Dubininella” and also describe two new species: “*Candidatus* Dubininella ochracea” and “*Candidatus* Gallionella aquifuscii”.

## 2. Results and Discussion

### 2.1. The Physicochemical Characteristics of the Sampling Site

The composition of the artesian mineral water from the Belaya Gorka spring is continuously monitored. Researchers note that the water’s properties have remained unchanged for many years. It is a cold chloride-type mineral water enriched with bromide, with a pH of 7.2 and a temperature of 11 °C. The iron content in the water at the sampling site reaches 0.5 mg/L; the bromide concentration varies from 36.8 to 48.9 mg/L; and the chloride content is 5745.6 mg/L. The water’s mineralization is within the range of 8.4–9.7 g/L. The water is characterized by slight carbonation with nitrogen and carbon dioxide [17]. Water of the Belaya Gorka type is very rare in nature. It may be a close analog of the mineral water of the Wiesbaden resort (Germany)—the “Three Lilies” spring.

### 2.2. An Overview of the Composition of the Microbial Community

The relative abundance of different taxonomic groups was estimated using SingleM v.0.21.3. This approach is based on searching for single-copy marker genes in shotgun metagenomic reads and their taxonomic classification using the GTDB [18].

**Figure 1:**
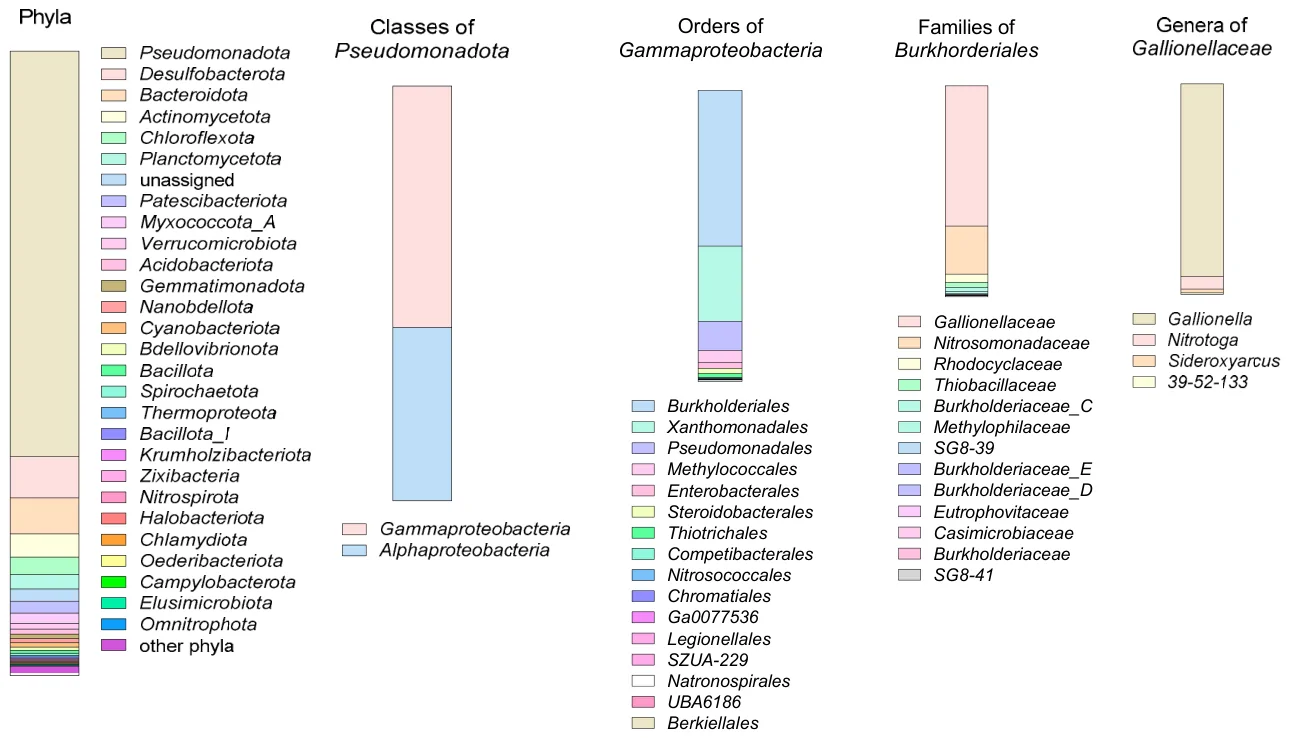
The taxonomic composition of the microbial community of ferruginous mat. The first column shows the relative proportions of individual phyla in the entire community; the second column shows the relative proportions of individual classes in the dominant phylum *Pseudomonadota*; the third column shows the relative proportions of individual orders in the dominant class *Gammaproteobacteria*; the fourth column shows the relative proportions of individual families in the dominant order *Burkhorderiales*; and the fifth column shows the relative proportions of individual genera in the dominant family *Gallionellaceae*. The taxonomy is shown according to the GTDB.

The dominant phylum was *Pseudomonadota* (65.0% of the metagenome), belonging to the classes *Alphaproteobacteria* (27.1%) and *Gammaproteobacteria* (37.8%). Among the *Gammaproteobacteria*, members of the order *Burkholderiales* predominated (20.1%), with the families *Gallionellaceae* (13.2%), *Nitrosomonadaceae* (4.6%), and *Rhodocyclaceae* (0.8%). The majority of *Gallionellaceae* belonged to the genus *Gallionella* (12.1%); representatives of *Nitrotoga*, *Sideroxyarcus, and the candidate genus* 39-52-133 were present at lower abundances (Figure 1).

### 2.3. Genome Assembly

From the sequenced metagenome, six MAGs (metagenome assembled genomes) assigned to the family *Gallionellaceae* were obtained. Two of them, designated BG-057 and BG-364, which met the standard for high-quality MAGs (>90% completeness and <5% contamination), were further analyzed (Table 1).

### 2.4. Taxonomic Placement

**Figure 2:**
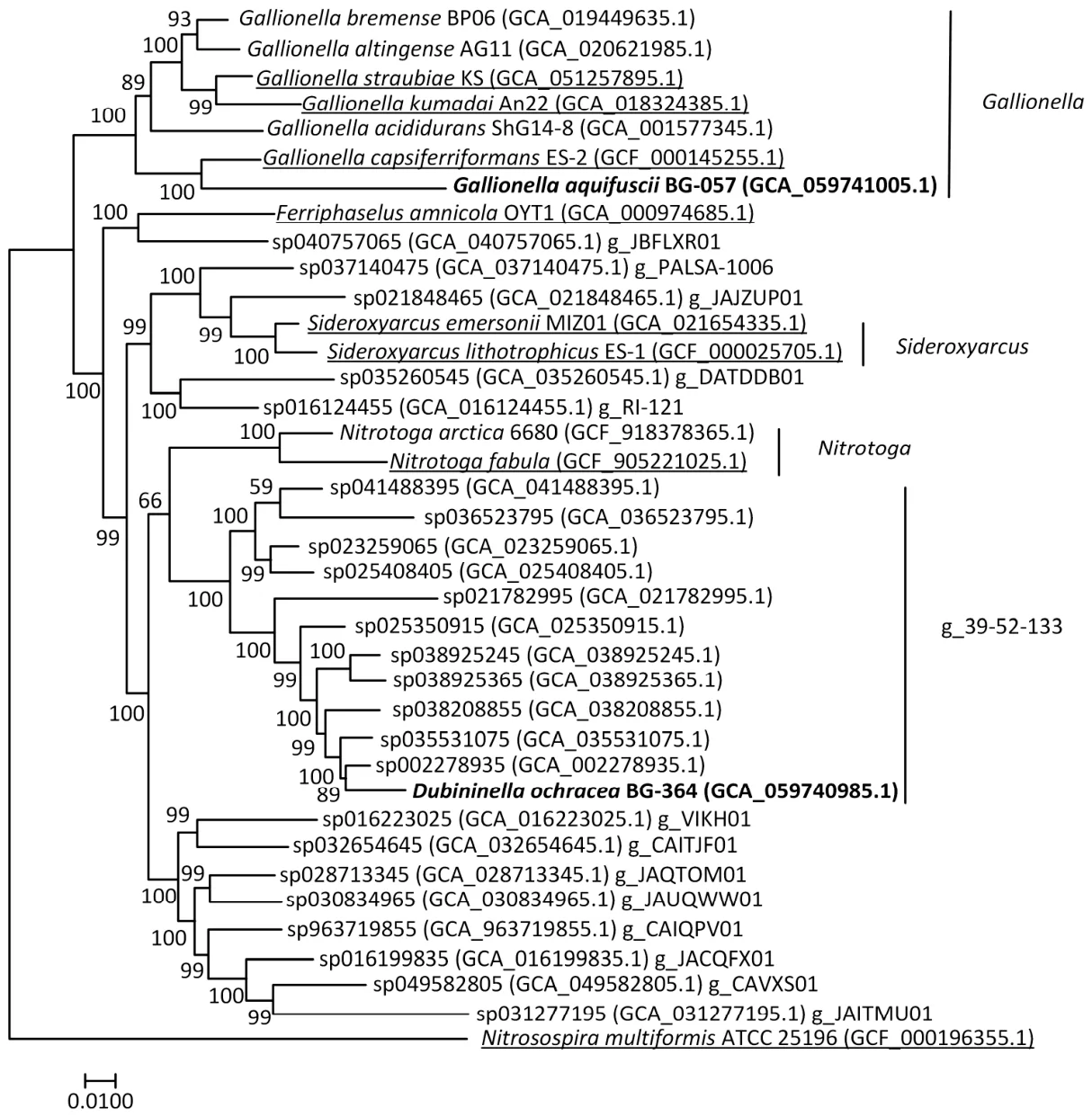
The genome-based phylogeny of the family *Gallionellaceae*. Taxonomic lineages are shown according to the GTDB release 232 (g, genus). Support for internal branches was calculated using the Bayesian test. The genome sequence of *Nitrosospira multiformis*, a member of the sister family *Nitrosomonadaceae*, was used as an outgroup. The genomes of cultured strains are underlined, and other genomes are MAGs.

On the phylogenetic tree constructed using concatenated amino acid sequences of 120 conserved proteins, BG-057 clusters with members of the genus *Gallionella*, whereas BG-364 clusters with representatives of a candidate genus designated as 39-52-133 in the GTDB (Figure 2).

ANI (average nucleotide identity) values between the BG-057 genome and other genomes of the genus *Gallionella* range from 73.95% to 79.69%, while those between BG-364 and other genomes of the genus 39-52-133 range from 75.07% to 79.15%. These values are below the generally accepted thresholds (95–96%) used for species delineation [19], indicating that BG-057 and BG-364 represent distinct species.

The AAI (average amino acid identity) value between BG-057 and its closest relative, *Gallionella capsiferriformans* ES-2, was 74.81%. This value is well above the previously proposed genus demarcation thresholds of 60–65% [20,21], confirming that BG-057 belongs to the genus *Gallionella*. Similarly, the AAI values between BG-364 and other genomes from the genus 39-52-133 were in the range from 70.44% to 79.30%, further supporting the assignment of BG-364 to this genus.

Thus, both the genome-based phylogeny and the genome similarity metrics support assigning BG-057 to a new species within the genus *Gallionella* (for which we propose the name “*Candidatus* Gallionella aquifuscii”) and strain BG-364 to a new species within the genus 39-52-133. We propose to assign the name “*Candidatus* Dubininella” to the genus 39-52-133 and describe the BG-364 lineage as a new species “*Candidatus* Dubininella ochracea”.

### 2.5. Iron Metabolism

Prokaryotes are able to use iron as an electron donor during lithotrophic growth, a terminal electron acceptor during anaerobic respiration, and as a common cofactor for many enzymes. Sequenced members of the *Gallionellaceae* family are able to oxidize ferrous iron on the cell surface, coupling this process with the ETC (electron transport chain), and transport iron into the cell, where it is used for biosynthetic purposes.

#### 2.5.1. Lithotrophic Growth in the Presence of Fe (II)

In 2023, a pan-genome analysis of 103 *Gallionellaceae* genomes demonstrated that all members of the family have the genetic potential for lithotrophic growth in the presence of Fe (II) or nitrite [7]. Most iron-oxidizing *Gallionellaceae* oxidize iron using outer membrane-localized cytochromes, which belong to the monoheme cytochrome Cyc2 (cluster 1) and decahem cytochrome MtoA. In addition, some iron-oxidizing *Gallionellaceae* possess the undeheme cytochrome Uet and multiheme cytochrome with a variable number of heme-binding sites PCC3 [7]. The sequenced genome of BG-364 encodes the monoheme cytochrome Cyc2 (cluster 1), while the genome of BG-057 encodes the decahem cytochrome MtoA and the monoheme cytochrome Cyc2 (cluster 1). Cyc2 is thought to oxidize dissolved Fe (II), while MtoA oxidizes insoluble Fe (II), which is present in oxides [22,23].

Both Cyc2 and MtoA are c-type cytochromes that transport electrons across the outer membrane. FeOB (iron-oxidizing bacteria) are known to utilize additional c-type cytochromes to transport electrons across the periplasm to the rest of the electron transport chain [7].

**Figure 3:**
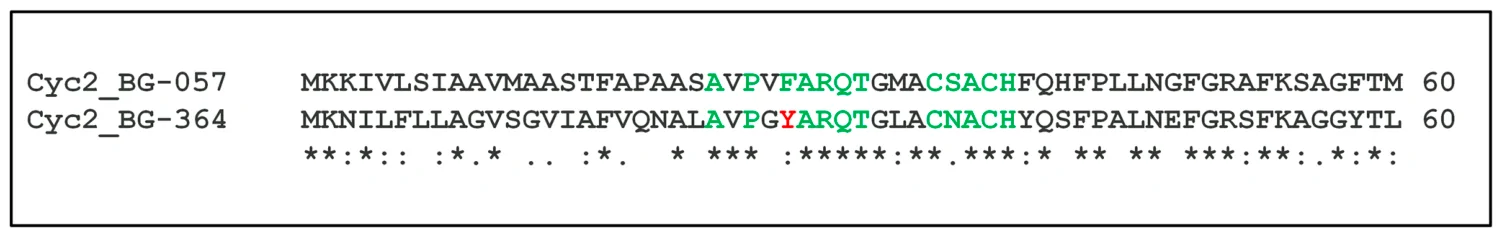
The alignment of the first 60 amino acids of Cyc2 from BG-057 and BG-364. The heme-binding site and conserved amino acids located upstream of the binding site are highlighted in green. The Y amino acid, which should be replaced by the F amino acid, is highlighted in red. Asterisk means complete identity; colon (:) means strong conservative substitution; dot (.) means weak conservative substitution.

The conserved AXPxFAR[Q/K][T/Y] motif, located five amino acids upstream of CXXCH, is known to be unique to Cyc2 and can serve as a diagnostic feature [24]. In the Cyc2 proteins identified in BG-364 and BG-057, this sequence is located three amino acids upstream of the CXXCH motif, and the F amino acid is replaced by a Y in BG-364 (Figure 3). A similar substitution and shift are found in some iron-oxidizing bacteria [25], but the functionality of Cyc2 proteins with such a substitution as iron oxidases has not been confirmed experimentally. 

Both genomes also encode Cyc1, which transports electrons from Cyc2 to other components of the ETC (Table S1).

The gene for the decahem cytochrome MtoA is absent from the BG-364 genome but is present in the BG-057 genome. MtoA proteins are structurally similar to MtrA proteins. MtoA oxidizes Fe (II) on the cell surface. MtrA is capable of reducing dissolved Fe (III) chelates located in the periplasm [26]. In [7], a tree was constructed based on MtoA from representatives of the *Gallionellaceae* family and MtrA from typical iron reducers. No clear clustering of MtoA and MtrA was observed. There is evidence that the electron flow through the Mto/Mtr systems may be reversible [27,28,29]. Thus, it cannot be ruled out that the functions of MtoA and MtrA are interchangeable and that, in essence, these are indistinguishable proteins capable of transferring electrons across the outer membrane in either direction [7].

It is known that the operon encoding MtrA in iron-reducing bacteria usually also encodes MtrC, an outer membrane cytochrome thought to be involved in dissimilatory iron reduction [30]. MtrC has been found only in iron-reducing bacteria and is not encoded by iron-oxidizing bacteria [31], supporting its use as an additional indicator of iron reduction potential [32]. MtrC was not detected in the BG-057 genome, which may indicate that the identified decahem cytochrome is the iron-oxidizing protein MtoA.

The same operon that encodes MtoA also encodes the porin MtoB and MtoD, a periplasmic monoheme cytochrome thought to mediate electron transfer between the external environment and the cytoplasm [33] (Table S1). This operon also contains a gene encoding octaheme cytochrome *c*, which has an N-terminal signal peptide. This cytochrome *c* may be localized in the periplasm and involved in electron transfer to the quinone pool or localized on the outer membrane and, along with MtoA, participates in Fe (II) oxidation.

BG-057, like many members of the family *Gallionellaceae* [7], encodes, in addition to the known iron oxidases, other multiheme cytochromes that can potentially participate in iron oxidation (Table S2). The BG-364 genome contains genes encoding multiheme cytochromes 6, 7, 11, and 24 hemes, which contain signal peptides (Table S3), indicating their localization in the periplasm or even on the outer membrane. These may also represent uncharacterized iron oxidases.

#### 2.5.2. Formation of Complexing Ligands Such as Siderophores and Transport of Fe–Siderophore Complexes into Cell

The formation of complexing ligands promotes the dissolution of solid-phase Fe (III) oxide, making soluble Fe (III) more accessible to the microorganism. Although these strategies have only been demonstrated for Fe (III)-reducing microorganisms, similar strategies may be used by Fe (II)-oxidizing microorganisms that utilize solid-phase Fe (II) electron donors [34].

High-affinity iron chelators called siderophores can act as such ligands [35]. Microorganisms can synthesize siderophores via the NRPS (non-ribosomal peptide synthetase) or NIS (NRPS-independent siderophore) pathways [36]. Many different siderophore synthesis pathways share homologous genes. Therefore, FeGenie cannot accurately predict which siderophore will be synthesized, but it allows for the identification of putative siderophore operons [32].

##### BG-057

The BG-057 genome contains genes that can serve as markers for the NRPS siderophore synthesis pathways VabF and Pchl, as well as a gene that can serve as a marker for the NIS siderophore synthesis pathway RhbA.

To export synthesized siderophores, diderm (Gram-stain-negative) bacteria most often use the PvdRT-OpmQ system—a multifunctional ABC transporter. It consists of three proteins that function as a single complex spanning both membranes of diderm bacteria:

PvdT—an intramembrane transporter and ATP-binding protein. Located in the inner membrane, it provides energy (through ATP hydrolysis) and forms a channel for siderophore passage.

PvdR—a periplasmic protein (periplasmic binding domain). Located in the periplasm, it functions as an “adapter,” binding the siderophore and transferring it to the PvdT transporter.

OpmQ—an outer membrane protein. It forms a channel in the outer membrane through which the siderophore is released.

The FeGenie program detects the presence of PvdRT genes. Both genes are found in the BG-057 genome, suggesting that it has the potential to transport synthesized siderophores to the outer membrane surface.

After the siderophore binds Fe (III), the resulting complex must be recognized by a receptor located on the cell surface and transported into the periplasm via active transport. In diderm bacteria, the most common transport mechanism is the Ton system (TonB-ExbB-ExbD protein complex) [35,37]. Because the Ton system can also transport other metabolites (e.g., vitamin B12), the identification of this transport pathway only suggests the potential for siderophore transport [32].

The BG-057 genome contains genes for the TonB-ExbB-ExbD complex but lacks genes for the Ton-dependent receptors PirA and PiuA, which are annotated by FeGenie. It is possible that another Ton-dependent receptor (several dozen are known [38]) is involved in recognizing the Fe (III)–siderophore complex, but it is not annotated by FeGenie.

Once the Fe (III)–siderophore complex reaches the periplasm, various mechanisms exist for its subsequent transport into the cytoplasm. The main pathway involves ABC transporters. The FepCDG system may be used for this purpose, but it is absent in BG-057.

In some cases, Fe (III) is first reduced to Fe (II) before being transported by an unknown ferroreductase in the periplasmic space. The resulting Fe (II) can then be transported across the inner membrane by the Feo system, the main components of which are FeoAB proteins. These proteins are found in the BG-057 genome. The FeoC transcriptional repressor has not been detected, but iron-regulating genes of the Fur family are present. The Feo system is similarly organized in the genomes of *Sideroxydans lithotrophicus* ES-1^T^ and *Gallionella capsiferriformans* ES-2^T^ [39].

Once in the cytoplasm, iron enters the general intracellular pool. From there, it is directed to iron–sulfur clusters and other iron-containing enzymes, and excess iron can be stored as a non-toxic mineral core within ferritin or bacterioferritin. The BG-057 genome encodes three ferritin-like domains. Ferritin proteins bind excess Fe (II) ions in the cytoplasm, oxidize them to Fe (III) using their ferroxidase activity, and store them in a non-toxic form within their hollow structure [40]. Such a mechanism can be assumed in representatives of the *Gallionellaceae* family based on the detection of ferritin-like proteins in many of them.

**Figure 4:**
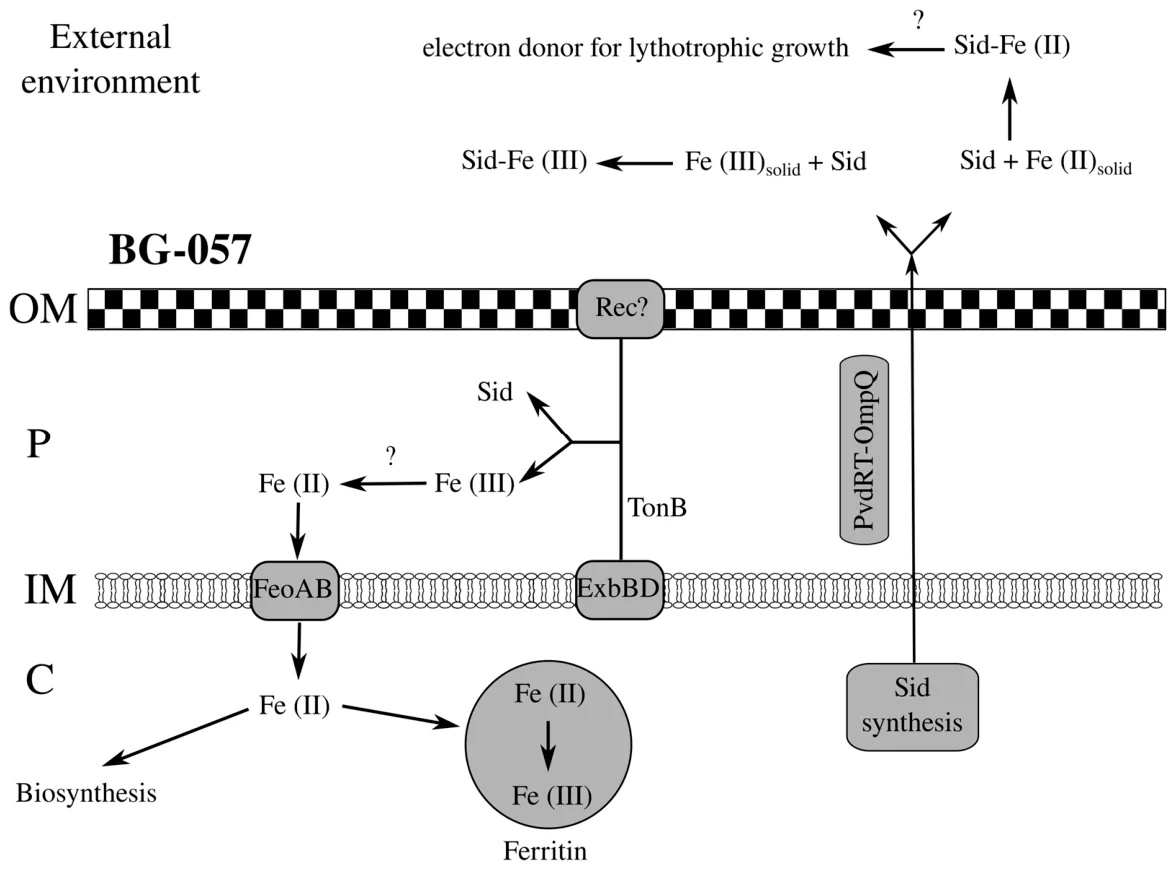
Scheme of assimilation of solid-phase iron by BG-057 bacteria. C is cytoplasm; IM is inner membrane; P is periplasm; OM is outer membrane; Sid is siderophore; PvdRT-OpmQ is multifunctional ABC transporter that transports siderophores to cell surface; Rec? is unknown Ton receptor; TonB is transmembrane proton channel; ExbBD is motor part of TonB-ExbB-ExbD complex, anchored in inner membrane; FeoAB is Feo system that transports Fe (II) from periplasm to cytoplasm; ? is unknown mechanism.

Since BG-057 has two iron oxidases, Cyc2 and MtoA, it is possible that the formation of the Fe (II)–siderophore complex will facilitate the availability of insoluble Fe (II) oxides for lithotrophic growth. However, iron can also be used for biosynthetic purposes. BG-057 synthesizes siderophores, transports them to the cell surface, then imports Fe (III) into the cell in a complex with siderophores, reduces the released Fe (III) to Fe (II) in the periplasm, and transports Fe (II) into the cytoplasm, where some Fe (II) is used for intracellular needs, while excess Fe is oxidized to Fe (III) and stored within the protein shell (Figure 4).

##### BG-364

The BG-364 genome lacks genes encoding siderophore biosynthesis pathways or the PvdRT-OpmQ system, which transports siderophores to the outer membrane. However, genes encoding the TonB-ExbB-ExbD complex and the Ton-dependent PirA receptor genes were identified. The presence of genes involved in the uptake of the Fe (III)–siderophore complex in the absence of genes encoding siderophore synthesis may be related to the fact that BG-364 coexists with BG-057 and other FeOB community members. Therefore, we can hypothesize that BG-364 may utilize siderophores secreted by other iron-oxidizing bacteria.

The BG-364 genome contains genes encoding FeoAB, which transports Fe (II) from the periplasm to the cytoplasm after Fe (III) reduction by an unknown ferroreductase. The FeoC transcriptional repressor was not detected, but iron-regulating genes such as Fur are present.

Ferritin-like domains are absent from the BG-364 genome, which may indicate that this organism is unable to store iron in the cytoplasm.

Therefore, it is presumed that BG-364 uses FeOB-synthesized siderophores to chelate Fe (III) on the cell surface, transports it to the periplasm, reduces it to Fe (II), transports it to the cytoplasm, and utilizes it entirely for biosynthetic purposes, without storing the excess.

### 2.6. Autotrophy

Both sequenced genomes encode all the genes necessary for autotrophic CO_2_ fixation via the CBB cycle, as well as genes for the auxiliary enzyme carbonic anhydrase. Both genomes contain genes for both type I and type II Rubisco (ribulose-1,5-bisphosphate carboxylase/oxygenase) (Table S1).

In the BG-057 genome, some of the genes encoding the CBB cycle enzymes are organized into two clusters, while the remaining genes are scattered individually across different regions of the genome. One cluster contains one copy of type I Rubisco, the regulatory genes for Rubisco, the gene for one of the CBB cycle enzymes, FBA II (fructose-1,6-bisphosphate aldolase), and the carbonic anhydrase gene. The other cluster encodes type II Rubisco, the regulatory genes for Rubisco, the genes encoding five CBB cycle enzymes, and the gene for the second copy of carbonic anhydrase. Another Rubisco gene colocalizes with carboxysome genes that function to concentrate carbon dioxide and enhance carbon fixation (Table S1).

In the BG-364 genome, the Rubisco type I and II genes are also encoded in a cluster of regulatory genes, while the genes for the remaining CBB cycle enzymes are located separately in different regions of the genome (Table S1).

### 2.7. Lithotrophy

#### 2.7.1. Lithotrophy in the Presence of Mn (II)

Manganese-oxidizing bacteria (MnOB) oxidize Mn(II) via enzymatic processes; most of the identified enzymes of this type belong to the multicopper-containing oxidases (MCOs) [41]. The gene for one of the MCOs that has been experimentally demonstrated to oxidize Mn(II) [41], McoA, was found in the genome of BG-364 (Table S1). In an aquatic environment, the oxidation of soluble Mn(II) to insoluble Mn(IV) (manganese oxides) occurs mainly via two pathways: biological and chemical oxidation. At pH values below 8.5, the biological process is significantly faster than the chemical one [42]. Since the pH of the sampled source was 7.2, the presence of *mcoA* suggests that BG-364 may oxidize Mn(II), thereby supporting lithotrophic growth.

The BG-057 genome lacks manganese oxidase genes.

#### 2.7.2. Lithotrophy in the Presence of Arsenic

The BG-364 genome encodes the large and small subunits of arsenite oxidase, AioA and AioB. This periplasmic enzymatic complex is capable of oxidizing As(III) to As(V), transferring electrons to the ETC [43]. Arsenite oxidation requires a two-component transcriptional regulatory system, including AioS (sensor histidine kinase) and AioR (transcriptional regulator) [44,45]. Both components of this system are encoded within a single operon with the arsenite oxidase subunits (Table S1).

#### 2.7.3. Lithotrophy in the Presence of Reduced Sulfur Compounds

Both sequenced genomes contain genes encoding enzymes for lithotrophic growth in the presence of reduced sulfur compounds. The two genomes encode different variants of hydrogen sulfide oxidation enzymes. The BG-057 genome encodes Sqr type IV. The BG-364 genome encodes Sqr type I. FCSD (flavocytochrome c sulfide dehydrogenase) genes were not detected in either genome. Furthermore, BG-364 is potentially capable of lithotrophic growth in the presence of thiosulfate, as its genome contains genes for the direct oxidation of thiosulfate to sulfate (SoxAXBYZCD). The BG-364 genome also contains genes for the oxidation of elemental sulfur to sulfite (the complete rDsr complex) and the oxidation of sulfite to sulfate (direct oxidation via SoeABC and indirect oxidation via APS through ATP sulfurylase and APS reductase) (Table S1).

Thus, BG-057 is potentially capable of lithotrophic growth through the oxidation of hydrogen sulfide, while BG-364 is capable of oxidizing hydrogen sulfide, thiosulfate, sulfite, and elemental sulfur.

#### 2.7.4. Hydrogenases

The BG-057 genome encodes the maturation proteins Hyp (HypABCDEF) and Hydrogenase_Group_3d (HoxFUYH) (Table S1). Group 3d hydrogenases are cytoplasmic, NAD-dependent, bidirectional enzymes involved in lithotrophic growth in the presence of molecular hydrogen. They oxidize hydrogen and reduce NAD to NADH [46]. The BG-364 genome lacks hydrogenase genes and genes encoding enzymes involved in their maturation. Based on the genomic data, BG-057 is potentially capable of lithotrophic growth in the presence of molecular hydrogen.

### 2.8. Nitrate Reduction

Both sequenced genomes contain genes for the assimilatory nitrate reductase NasA. Both genomes encode genes for the assimilatory nitrite reductase NirBD, which reduces nitrite to ammonium. Thus, both organisms are potentially capable of assimilatory nitrate reduction.

Genes encoding the dissimilatory nitrate reductase NarGHI are found only in the BG-364 genome. This genome also contains the gene for the dissimilatory nitrite reductase NirS, which produces NO. Both genomes also contain genes encoding the nitric oxide reductase NorBC, which reduces NO to N_2_O. The genes for the nitrous oxide reductase NosZ are absent from both genomes. Thus, BG-364 is potentially capable of denitrification to N_2_O (Table S1).

Nitrate and nitrite can form in the aerobic zone of the spring as a result of ammonium oxidation by nitrifying bacteria. Nitrifiers of both the first group (e.g., *Nitrosomonas*) and the second group (e.g., *Nitrotoga*) were detected in the community.

### 2.9. Terminal Oxidases and Reverse Electron Transport

The BG-057 genome encodes genes for three types of terminal oxidases: bd-type quinol oxidase (Cyd), cbb_3_-type cytochrome *c* oxidase (Cco), and heme–copper oxidase (Table S1). The latter is likely a type aa_3_ cytochrome *c* oxidase, as indicated by the presence of the PF00115 and PF00116 domains in subunits 1 and 2, respectively. Cbb_3_- and bd-type oxidases are known to be high-affinity terminal oxygen reductases capable of functioning under microaerobic conditions [47,48]. The highest affinity for oxygen (Km from 3 to 8 nM) has been reported for the bd-type oxidase [49]. Cytochrome *c* oxidases of the aa_3_ type have low affinity for oxygen and function as terminal oxidases in the respiratory chain of aerobic prokaryotes [50]. The BG-057 genome lacks genes encoding the major reductases required for anaerobic respiration. However, the organism possesses multiple terminal oxidases that may support aerobic respiration under different oxygen concentrations, with their relative contributions potentially depending on their oxygen affinities.

The BG-364 genome contains the aerobic terminal oxidases cbb3 (Cco) and aa3-type (Cox) (Table S1). Since the BG-364 genome also contains denitrification genes, it can be assumed that in the presence of oxygen, this organism respires aerobically, and in the absence of oxygen, it may switch to anaerobic respiration using nitrate.

Members of the family *Gallionellaceae* possess genes for cytochrome bc_1_ and the quinol oxidase complex of ACIII (alternative complex III), which direct electrons to the quinone pool, where they can be used to form NADH for biosynthetic reactions of the CBB cycle. The presence of bc_1_ and ACIII often occurs in the same organism, contributing to the flexibility of the electron transport chains in *Gallionellaceae* [7]. The genome of BG-057 encodes the bc_1_ complex and ACIII. The genome of BG-364 lacks genes for ACIII, but a fusion protein containing the cytochrome *b* and *c* domains, presumably the bc_1_ complex, is encoded at the boundary of a short contig (Table S1). Thus, we can hypothesize that during lithoautotrophic growth, BG-057 mediates reverse electron transport via cytochrome bc_1_ and ACIII, while BG-364 mediates reverse electron transport only via cytochrome bc_1_.

Both genomes also encode an Rnf complex (RnfABCDGE). In *Gallionellaceae*, it is a membrane-bound, ion-translocating electron transport enzyme (ferredoxin:NAD^+^ oxidoreductase) [7]. The complex acts as a metabolic bridge, reversibly transferring electrons between NADH and low-potential ferredoxin while utilizing a transmembrane ion gradient to drive the reactions. Under certain conditions, the complex functions in reverse, oxidizing reduced ferredoxin and reducing NAD^+^ to pump ions (Na^+^ or H^+^) out of the cell, building a chemiosmotic gradient that helps produce ATP.

### 2.10. Central Metabolism

The BG-057 genome contains the complete set of genes required for glycolysis. The BG-364 genome lacks the glucokinase and hexokinase genes, which convert glucose to glucose-6-phosphate. However, glucose-6-phosphate can be formed via gluconeogenesis and glycogenolysis. The BG-364 genome encodes all the enzymes needed for this (Table S1).

Both sequenced genomes encode genes for the oxidative pentose phosphate pathway (OPPP). Pyruvate, formed through glycolysis and the OPPP, is metabolized to acetyl-CoA in the two sequenced organisms via different pathways: in BG-057 via pyruvate:ferredoxin oxidoreductase (PFOR) and pyruvate dehydrogenase complex and in BG-364 via the PFOR complex. Although it has traditionally been believed that PFOR can function only under anaerobic conditions [51], the ability of PFOR to function in the presence of oxygen is known for some organisms [52]. During operation, PFOR supplies the cell with reduced ferredoxin, which can be used in other reactions requiring low-potential electrons. Therefore, we can assume that PFOR is capable of functioning in BG-057 at low oxygen concentrations, while at high oxygen concentrations, the pyruvate dehydrogenase complex is more likely to function. Acetyl-CoA then enters the TCA (tricarboxylic acid) cycle, for which the full set of genes was identified in both genomes (Table S1).

### 2.11. Description of New Genus and Species

#### 2.11.1. Description of “*Candidatus* Dubininella”

Etymology: Du.bi.ni.nel’la. N.L. fem. dim. n. Dubininella, named for G.A. Dubinina, a Russian microbiologist who made major contributions to the study of iron bacteria.

Representatives of this genus are potentially capable of lithotrophic growth in the presence of Fe (II), Mn (II), As (III), and reduced sulfur compounds. They are potentially capable of aerobic and anaerobic respiration (incomplete denitrification), autotrophic growth via the CBB cycle, and heterotrophic growth via glycolysis, OPPP, and the TCA cycle.

#### 2.11.2. Description of “*Candidatus* Dubininella ochracea”

Etymology: o.chra’ce.a. N.L. fem. adj. ochracea, like ocher; from L. fem. n. ochra, yellow-ocher.

Not cultivated. Cells presumably have a respiratory type of metabolism and are facultative anaerobes. They are potentially capable of denitrification under anaerobic conditions (from nitrate to N_2_O). They are potentially capable of lithoautotrophic and mixotrophic growth. During lithotrophic growth, they obtain energy by oxidizing Fe (II), As (III), Mn (II) and reduced sulfur compounds. They are potentially capable of carbon fixation through the CBB cycle.

Source: ferruginous spring.

The species is represented by MAG BG-364, obtained from the metagenome of bacterial fouling formed in water flowing from the Belaya Gorka ferruginous spring, Russia.

GC fraction of genomic DNA (%): 49.6 (genome sequence).

GenBank accession number (whole genome assembly): JCAWNS000000000.

#### 2.11.3. Description of “*Candidatus* Gallionella aquifuscii”

Etymology: a.qui.fus’ci.i L. fem. n. aqua, water; L. masc. adj. fuscus, brown; N.L. gen. masc. adj. aquifuscii, pertaining to brown water.

Not cultivated. Cells presumably have a respiratory type of metabolism and are facultative lithoautotrophs. During lithoautotrophic growth, they obtain energy by oxidizing Fe (II), H_2_, and H_2_S and fixing carbon in the CBB. They are potentially capable of heterotrophic growth.

Source: ferruginous spring.

The species is represented by MAG BG-057, obtained from the metagenome of bacterial fouling formed in water flowing from the Belaya Gorka ferruginous spring, Russia.

GC fraction of genomic DNA (%): 53.1 (genome sequence).

GenBank accession number (whole genome assembly): JCAWNT000000000.

The main characteristics of “*Candidatus* Dubininella ochracea” and “*Candidatus* Gallionella aquifuscii” are given in Table S4.

## 3. Materials and Methods

### 3.1. Sampling

**Figure 5:**
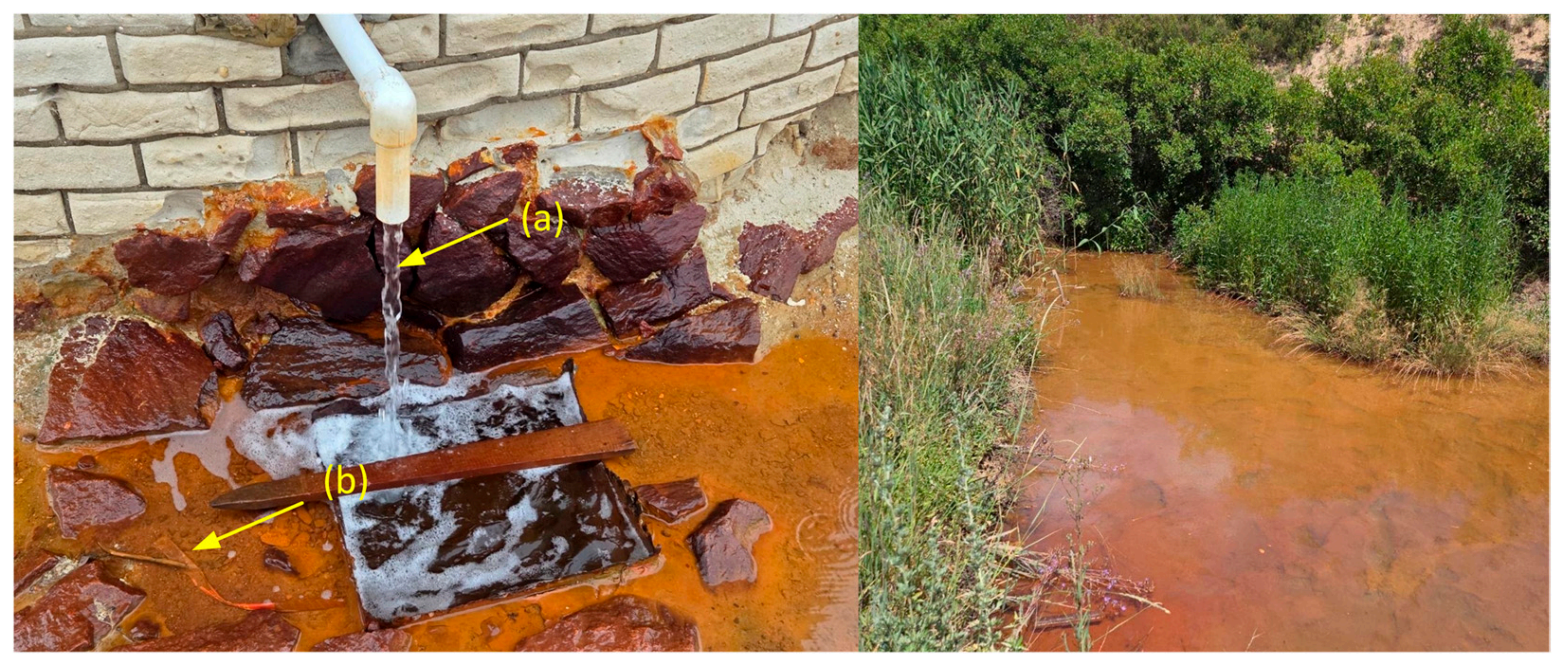
Photographs of the sampling site. The arrow indicates the location of water sampling for chemical analysis (a) and the location of microbial mat sampling for metagenomic analysis (b).

Water samples for chemical analysis and microbial mat samples for molecular studies were collected on 20 June 2025 from the surface outflow of an artesian mineral water spring near the Belaya Gorka sanatorium, Voronezh region, Russia (49°47′33″ N, 40°57′7″ E). Photographs of the sampling site are shown in Figure 5.

### 3.2. Metagenome Sequencing and MAG Assembly

Total DNA from the microbial mat sample was isolated using the DNeasy PowerMax Soil Kit (Qiagen, Hilden, Germany). GeneMind (GeneMind Biosciences, Shenzhen, China) high-throughput sequencing technology, which is analogous to Illumina technology, was used for metagenome sequencing. A shotgun library was prepared using the Nextera DNA Library Preparation Kit (Illumina, San Diego, CA, USA) and sequenced on a FastaSeq300 (GeneMind Biosciences) instrument using an FCX flow cell (GeneMind Biosciences) in a paired-end read format (2 × 300 nucleotides). A total of 137,378,255 read pairs with a total yield of 82.4 Gb were obtained. Adapter sequences in the obtained reads were removed using Cutadapt v.4.8 [53], and low-quality regions (Q < 30) were removed using Sickle v. 1.33. Overlapping reads were merged using FLASH v.1.2.11 [54]. The obtained sequences were assembled into contigs using MEGAHIT v.1.2.9 [55]. The assembled contigs were binned into metagenome-assembled genomes (MAGs) using the programs MetaBAT v.2.15 [56], COMEBin v.1.0.4 [57], Concoct v.1.1.0 [58], and MaxBin2 v.2.2.7 [59]. The binning results were combined into an optimized set of MAGs using DAS Tool v.1.1.7 [60].

The completeness and contamination of the MAGs were assessed using CheckM2 v.1.0.2 [61]. The taxonomic identification of the MAGs was carried out using the GTDB-Tk v.2.4.1 tool [62] and the GTDB release 232 [10].

The relative abundance of different taxonomic groups in the metagenome was assessed using SingleM v.0.21.3 [18]. A random selection of 50 million reads was used.

### 3.3. Genome Analysis

Genes were identified and annotated using RAST [63], after which the annotation was manually corrected by comparing the predicted amino acid sequences with the NCBI databases. Signal P v.5.0 [64] was used to predict N-terminal signal peptides, and transmembrane domains were identified using TMHMM Server v.2.0 [65]. Iron metabolism genes were searched using the FeGenie bioinformatics tool [32].

A total of 120 single-copy marker genes were identified in the analyzed genomes, concatenated, and merged with the reference GTDB alignment of 189,842 bacterial genomes using the GTDB-Tk v.2.4.1 pipeline. Columns with >50% gaps or with a single amino acid spanning <25% or >95% of taxa in the reference GTDB alignment were removed. The alignment was trimmed to ~5000 a.a. in length, with 42 random a.a. for each marker gene as described in the GTDB methods (https://gtdb.ecogenomic.org/methods accessed on 23 August 2026). PhyML v. 3.3 [66] was used to construct the maximum-likelihood phylogenetic tree. The default parameters were used: the LG substitution model and four discrete rate categories with an estimated gamma shape parameter. Branch support values were estimated using the aBayes (Approximate Bayes) algorithm [67]. *Nitrosospira multiformis*, a member of the sister family *Nitrosomonadaceae*, was used as an outgroup.

## 4. Conclusions

**Figure 6:**
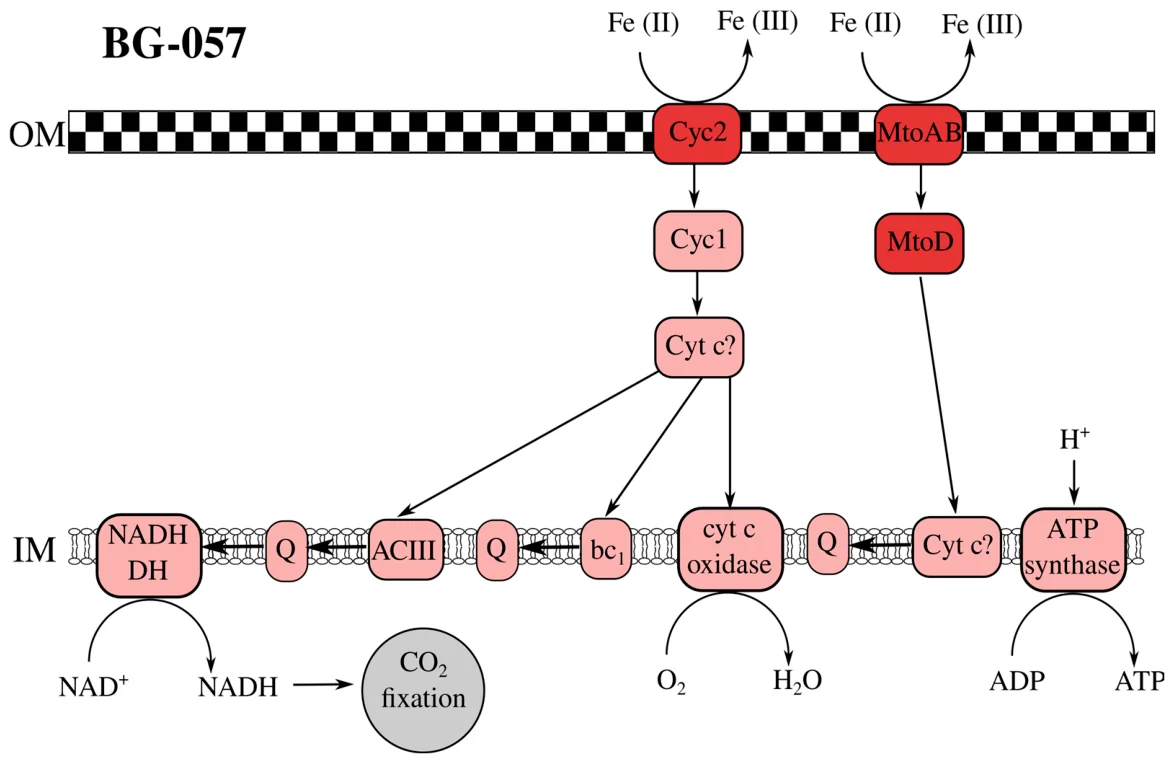
Potential electron transfer pathways for BG-057 during lithoautotrophic growth in the presence of Fe (II). MtoAB is a complex of a decaheme cytochrome and a porin involved in the oxidation of Fe(II), Cyc2 is a monoheme cytochrome involved in the oxidation of Fe(II), Cyc1 is a periplasmic diheme cytochrome involved in electron transfer, MtoD is a periplasmic monoheme cytochrome involved in electron transfer, Cyt c? is an unknown cytochrome c involved in electron transfer, NADH DH is the NADH dehydrogenase complex, Q is the quinone pool, bc_1_ is the bc_1_ complex, AC III is alternative complex III, cyt c oxidases are terminal cytochrome c oxidases, OM is the outer membrane, and IM is the inner membrane.

**Figure 7:**
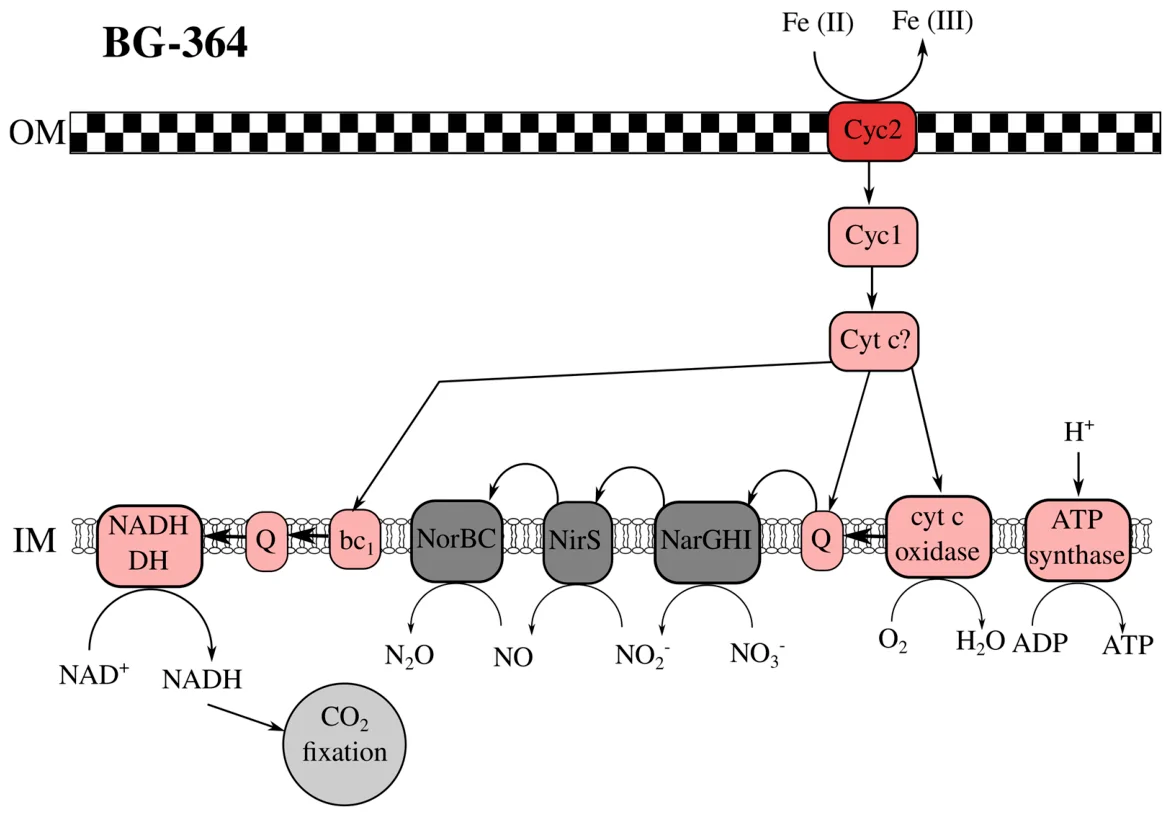
Potential electron transfer pathways in BG-364 during aerobic and anaerobic lithotrophic growth in the presence of Fe (II). Cyc2 is a monoheme cytochrome involved in Fe (II) oxidation, Cyc1 is a periplasmic diheme cytochrome involved in electron transfer, Cyt c? is an unknown cytochrome c involved in electron transfer, NADH DH is the NADH dehydrogenase complex, Q is a quinone pool, bc_1_ is the bc_1_ complex, cyt c oxidases are terminal cytochrome c oxidases, NarGHI is nitrate reductase, NirS is nitrite reductase, NorBC is NO reductase, OM is an outer membrane, and IM is an inner membrane.

For all iron-oxidizing bacteria from the family *Gallionellaceae*, the primary pathway for energy production is lithotrophic growth in the presence of Fe (II). Both *Gallionellaceae* representatives sequenced in this study are potentially capable of oxidizing Fe (II): BG-364 oxidizes Fe (II) via Cyc2, while BG-057 oxidizes Fe (II) via Cyc2 and MtoA (Figure 6 and Figure 7). However, the metabolic potential of the two organisms differs significantly in other respects.

In addition to lithotrophic growth in the presence of iron, BG-057 is potentially capable of lithotrophic growth in the presence of molecular hydrogen due to the presence of group 3d hydrogenase in its genome and lithotrophic growth in the presence of sulfide due to the presence of type IV Sqr. It is potentially capable of autotrophic growth via the CBB cycle, and during lithoautotrophic growth, it performs reverse electron transport via the bc_1_ complex and ACIII. It is potentially capable of aerobic respiration involving high-affinity terminal oxidases of the bd and cbb_3_ types for oxygen. It is potentially capable of heterotrophic growth.

In addition to lithotrophic growth in the presence of iron, strain BG-364 is potentially capable of lithotrophic growth in the presence of arsenite via arsenite oxidase AioAB, Mn(II) via McoA, sulfide via Sqr type I, thiosulfate via the SoxAXBYZCD system, elemental sulfur (due to the complete rDsr complex), and sulfite, both by direct oxidation (via SoeABC) and by indirect oxidation via APS (via ATP sulfurylase and APS reductase). It is potentially capable of aerobic respiration involving terminal oxidases cbb_3_ or aa_3_, and under oxygen deficiency, it is potentially capable of denitrification to N_2_O. It is also potentially capable of autotrophic growth via the CBB cycle and mixotrophic growth, similarly to some members of the family *Gallionellaceae* [68,69,70].

## Figures and Tables

**Table 1:** High-quality MAGs of the family *Gallionellaceae*.

Genome No.	Genome Assembly	Size (Mb)	Contigs	Completeness (%)	Contamination (%)	G + C Content (Mol%)
BG-057	JCAWNT000000000	3.55	209	93.24	1.67	53.1
BG-364	JCAWNS000000000	3.17	333	96.71	1.95	49.6

## Data Availability

The original contributions presented in this study are included in the article/Supplementary Material. Further inquiries can be directed to the corresponding authors.

## Supplementary Materials

The following supporting information can be downloaded at https://www.mdpi.com/article/10.3390/ijms27198801/s1.

## Abbreviations

The following abbreviations are used in this manuscript:

ANI	Average nucleotide identity
AAI	Average amino acid identity
CBB cycle	Calvin-Benson-Bassham cycle
ATP	Adenosine triphosphate
APS	Adenosine 5′-phosphosulfate
MAG	Metagenome-assembled genome
NADH	Nicotinamide adenine dinucleotide
GTDB	Genome Taxonomy Database
ETC	Electron transport chain
FeOB	Iron-oxidizing bacteria
NRPS	Non-ribosomal peptide synthetase
NIS	NRPS-independent siderophore
ABC	ATP-binding cassette
Rubisco	Ribulose-1,5-bisphosphate carboxylase/oxygenase
FCSD	Flavocytochrome c sulfide dehydrogenase
FBA	Fructose-1,6-bisphosphate aldolase
Km	Michaelis constant
ACIII	Alternative complex III
OPPP	Oxidative pentose phosphate pathway
PFOR	Pyruvate:ferredoxin oxidoreductase
TCA	Tricarboxylic acid
NCBI	National Center for Biotechnology Information

## References

[1] Ehrenberg G. (1836). Vorlaufige Mitteilung iiber das wirkliche Vorkommen fossiler Infusorien und ihre grosse Verbreitung. Ann. Phys..

[2] Winogradsky S.J. (1888). Über Eisenbakterien. Bot. Ztg..

[3] Winogradsky S. (1922). Eisenbakterien als Anorgoxydanten. Zentralblatt Für Bakteriol. Parasitenkd. Und Infekt..

[4] Hallbeck L.E.-L., Pedersen K., Whitman W.B. (2015). Gallionella. Bergey’s Manual of Systematics of Archaea and Bacteria.

[5] Huang Y.M., Jakus N., Straub D., Konstantinidis K.T., Blackwell N., Kappler A., Kleindienst S. (2022). ‘*Candidatus* ferrigenium straubiae’ sp. nov., ‘*Candidatus* ferrigenium bremense’ sp. nov., ‘*Candidatus* ferrigenium altingense’ sp. nov., are autotrophic Fe(II)-oxidizing bacteria of the family *Gallionellaceae*. Syst. Appl. Microbiol..

[6] Chan C.S., Fakra S.C., Emerson D., Fleming E.J., Edwards K.J. (2011). Lithotrophic iron-oxidizing bacteria produce organic stalks to control mineral growth: Implications for biosignature formation. ISME J..

[7] Hoover R.L., Keffer J.L., Polson S.W., Chan C.S. (2023). *Gallionellaceae* pangenomic analysis reveals insight into phylogeny, metabolic flexibility, and iron oxidation mechanisms. mSystems.

[8] Huang Y.M., Straub D., Blackwell N., Kappler A., Kleindienst S. (2021). Meta-omics reveal *Gallionellaceae* and *Rhodanobacter* species as interdependent key players for Fe(II) oxidation and nitrate reduction in the autotrophic enrichment culture KS. Appl. Environ. Microbiol..

[9] Uchijima T., Kato S., Tanimoto K., Shiraishi F., Hamamura N., Tokunaga K., Makita H., Kondo M., Ohkuma M., Mitsunobu S. (2025). Custom-made medium approach for effective enrichment and isolation of chemolithotrophic iron-oxidizing bacteria. FEMS Microbiol. Ecol..

[10] Parks D.H., Chuvochina M., Waite D.W., Rinke C., Skarshewski A., Chaumeil P.A., Hugenholtz P. (2018). A standardized bacterial taxonomy based on genome phylogeny substantially revises the tree of life. Nat. Biotechnol..

[11] Khalifa A., Nakasuji Y., Saka N., Honjo H., Asakawa S., Watanabe T. (2018). *Ferrigenium kumadai* gen. nov., sp. nov., a microaerophilic iron-oxidizing bacterium isolated from a paddy field soil. Int. J. Syst. Evol. Microbiol..

[12] Emerson D., Rentz J.A., Lilburn T.G., Davis R.E., Aldrich H., Chan C., Moyer C.L. (2007). A novel lineage of proteobacteria involved in formation of marine Fe-oxidizing microbial mat communities. PLoS ONE.

[13] Nunley J.W., Krieg N.R. (1968). Isolation of *Gallionella ferruginea* by use of formalin. Can. J. Microbiol..

[14] Emerson D., Moyer C. (1997). Isolation and characterization of novel iron-oxidizing bacteria that grow at circumneutral pH. Appl. Environ. Microbiol..

[15] Kato S., Krepski S., Chan C., Itoh T., Ohkuma M. (2014). *Ferriphaselus amnicola* gen. nov., sp. nov., a neutrophilic, stalk-forming, iron-oxidizing bacterium isolated from an iron-rich groundwater seep. Int. J. Syst. Evol. Microbiol..

[16] Kadnikov V.V., Ivasenko D.A., Beletskii A.V., Mardanov A.V., Danilova E.V., Pimenov N.V., Karnachuk O.V., Ravin N.V. (2016). A novel uncultured bacterium of the family *Gallionellaceae*: Description and genome reconstruction based on the metagenomic analysis of microbial community in acid mine drainage. Microbiology.

[17] Zhiskar M. (2022). Bromine and iodine-bromine mineral waters of the Voronezh region. Vestn. Voronezhskogo Gos. Univ. Seriya Geogr. Geoekologiya.

[18] Woodcroft B.J., Aroney S.T.N., Zhao R., Cunningham M., Mitchell J.A.M., Nurdiansyah R., Blackall L., Tyson G.W. (2026). Comprehensive taxonomic identification of microbial species in metagenomic data using SingleM and Sandpiper. Nat. Biotechnol..

[19] Konstantinidis K.T., Tiedje J.M. (2005). Genomic insights that advance the species definition for prokaryotes. Proc. Natl. Acad. Sci. USA.

[20] Konstantinidis K.T., Rosselló-Móra R., Amann R. (2017). Uncultivated microbes in need of their own taxonomy. ISME J..

[21] Riesco R., Trujillo M.E. (2024). Update on the proposed minimal standards for the use of genome data for the taxonomy of prokaryotes. Int. J. Syst. Evol. Microbiol..

[22] Zhou N., Kupper R.J., Catalano J.G., Thompson A., Chan C.S. (2022). Biological oxidation of Fe(II)-bearing smectite by microaerophilic iron oxidizer *Sideroxydans lithotrophicus* using dual Mto and Cyc2 iron oxidation pathways. Environ. Sci. Technol..

[23] Keffer J.L., Zhou N., Rushworth D.D., Yu Y., Chan C.S. (2025). Microbial magnetite oxidation via MtoAB porin-multiheme cytochrome complex in *Sideroxydans lithotrophicus* ES-1. Appl. Environ. Microbiol..

[24] Keffer J.L., McAllister S.M., Garber A.I., Hallahan B.J., Sutherland M.C., Rozovsky S., Chan C.S. (2021). Iron oxidation by a fused cytochrome-porin common to diverse iron-oxidizing bacteria. mBio.

[25] He S., Barco R.A., Emerson D., Roden E.E. (2017). Comparative genomic analysis of neutrophilic iron(II) oxidizer genomes for candidate genes in extracellular electron transfer. Front. Microbiol..

[26] Pitts K.E., Dobbin P.S., Reyes-Ramirez F., Thomson A.J., Richardson D.J., Seward H.E. (2003). Characterization of the *Shewanella oneidensis* MR-1 decaheme cytochrome MtrA: Expression in *Escherichia coli* confers the ability to reduce soluble Fe(III) chelates. J. Biol. Chem..

[27] Liu J., Wang Z., Belchik S.M., Edwards M.J., Liu C., Kennedy D.W., Merkley E.D., Lipton M.S., Butt J.N., Richardson D.J. (2012). Identification and characterization of MtoA: A decaheme C-type cytochrome of the neutrophilic Fe(II)-oxidizing bacterium *Sideroxydans lithotrophicus* ES-1. Front. Microbiol..

[28] Ross D.E., Flynn J.M., Baron D.B., Gralnick J.A., Bond D.R. (2011). Towards electrosynthesis in Shewanella: Energetics of reversing the Mtr pathway for reductive metabolism. PLoS ONE.

[29] Jain A., Kalb M.J., Gralnick J.A. (2022). Reconstructing electron transfer components from an Fe(II)-oxidizing bacterium. Microbiology.

[30] Lower B.H., Shi L., Yongsunthon R., Droubay T.C., McCready D.E., Lower S.K. (2007). Specific bonds between an iron oxide surface and outer membrane cytochromes MtrC and OmcA from *Shewanella oneidensis* MR-1. J. Bacteriol..

[31] Shi L., Fredrickson J.K., Zachara J.M. (2014). Genomic analyses of bacterial porin-cytochrome gene clusters. Front. Microbiol..

[32] Garber A.I., Nealson K.H., Okamoto A., McAllister S.M., Chan C.S., Barco R.A., Merino N. (2020). FeGenie: A comprehensive tool for the identification of iron genes and iron gene neighborhoods in genome and metagenome assemblies. Front. Microbiol..

[33] Coelho A., Silva J.M., Cantini F., Piccioli M., Louro R.O., Paquete C.M. (2024). Resonance assignments of cytochrome MtoD from the extracellular electron uptake pathway of *Sideroxydans lithotrophicus* ES-1. Biomol. NMR Assign..

[34] Weber K.A., Achenbach L.A., Coates J.D. (2006). Microorganisms pumping iron: Anaerobic microbial iron oxidation and reduction. Nat. Rev. Microbiol..

[35] Krewulak K.D., Vogel H.J. (2011). TonB or not TonB: Is that the question?. Biochem Cell Biol..

[36] Carroll C.S., Moore M.M. (2018). Ironing out siderophore biosynthesis: A review of non-ribosomal peptide synthetase (NRPS)-independent siderophore synthetases. Crit. Rev. Biochem Mol. Biol..

[37] Contreras H., Chim N., Credali A., Goulding C.W. (2014). Heme uptake in bacterial pathogens. Curr. Opin. Chem. Biol..

[38] Luscher A., Moynié L., Auguste P.S., Bumann D., Mazza L., Pletzer D., Naismith J.H., Köhler T. (2018). TonB-Dependent receptor repertoire of *Pseudomonas aeruginosa* for uptake of siderophore-drug conjugates. Antimicrob. Agents Chemother..

[39] Emerson D., Field E.K., Chertkov O., Davenport K.W., Goodwin L., Munk C., Nolan M., Woyke T. (2013). Comparative genomics of freshwater Fe-oxidizing bacteria: Implications for physiology, ecology, and systematics. Front. Microbiol..

[40] Bradley J.M., Le Brun N.E., Moore G.R. (2016). Ferritins: Furnishing proteins with iron. J. Biol. Inorg. Chem..

[41] Guo Y., Liu Y., Li F., Pan H., Yi X., Liu Y., Zhou H. (2026). Heterologous expression, purification and characteristics of a high molecular weight manganese oxidase McoA from *Pseudomonas* sp. AN-1. Int. J. Biol. Macromol..

[42] He B., Yun Z., Shi J., Jiang G. (2013). Research progress of heavy metal pollution in China: Sources, analytical methods, status, and toxicity. Chin. Sci. Bull..

[43] Yan G., Chen X., Du S., Deng Z., Wang L., Chen S. (2019). Genetic mechanisms of arsenic detoxification and metabolism in bacteria. Curr. Genet.

[44] Kashyap D.R., Botero L.M., Franck W.L., Hassett D.J., McDermott T.R. (2006). Complex regulation of arsenite oxidation in *Agrobacterium tumefaciens*. J. Bacteriol..

[45] Koechler S., Cleiss-Arnold J., Proux C., Sismeiro O., Dillies M.A., Goulhen-Chollet F., Hommais F., Lièvremont D., Arsène-Ploetze F., Coppée J.Y. (2010). Multiple controls affect arsenite oxidase gene expression in *Herminiimonas arsenicoxydans*. BMC Microbiol..

[46] Vignais P.M. (2008). Hydrogenases and H^+^-reduction in primary energy conservation. Results Probl. Cell Differ..

[47] Fang Y., Liu J., Yang J., Wu G., Hua Z., Dong H., Hedlund B.P., Baker B.J., Jiang H. (2022). Compositional and metabolic responses of autotrophic microbial community to salinity in lacustrine environments. mSystems.

[48] Bueno E., Mesa S., Bedmar E.J., Richardson D.J., Delgado M.J. (2012). Bacterial adaptation of respiration from oxic to microoxic and anoxic conditions: Redox control. Antioxid. Redox Signal.

[49] D’mello R., Hill S., Poole R.K. (1996). The cytochrome bd quinol oxidase in *Escherichia coli* has an extremely high oxygen affinity and two oxygen-binding haems: Implications for regulation of activity in vivo by oxygen inhibition. Microbiology.

[50] Lyons J., Aragão D., Slattery O., Pisliakov A.V., Soulimane T., Caffrey M. (2012). Structural insights into electron transfer in caa3-type cytochrome oxidase. Nature.

[51] Pan N., Imlay J.A. (2001). How does oxygen inhibit central metabolism in the obligate anaerobe Bacteroides thetaiotaomicron. Mol. Microbiol..

[52] Wang Y., Chen X., Spengler K., Terberger K., Boehm M., Appel J., Barske T., Timm S., Battchikova N., Hagemann M. (2022). Pyruvate:Ferredoxin oxidoreductase and low abundant ferredoxins support aerobic photomixotrophic growth in cyanobacteria. Elife.

[53] Martin M. (2011). Cutadapt removes adapter sequences from high-throughput sequencing reads. EMBnet. J..

[54] Magoč T., Salzberg S.L. (2011). FLASH: Fast length adjustment of short reads to improve genome assemblies. Bioinformatics.

[55] Li D., Liu C.M., Luo R., Sadakane K., Lam T.W. (2015). MEGAHIT: An ultra-fast single-node solution for large and complex metagenomics assembly via succinct de Bruijn graph. Bioinformatics.

[56] Kang D.D., Li F., Kirton E., Thomas A., Egan R., An H., Wang Z. (2019). MetaBAT 2: An adaptive binning algorithm for robust and efficient genome reconstruction from metagenome assemblies. PeerJ.

[57] Wang Z., You R., Han H., Liu W., Sun F., Zhu S. (2024). Effective binning of metagenomic contigs using contrastive multi-view representation learning. Nat. Commun..

[58] Alneberg J., Bjarnason B.S., de Bruijn I., Schirmer M., Quick J., Ijaz U.Z., Lahti L., Loman N.J., Andersson A.F., Quince C. (2014). Binning metagenomic contigs by coverage and composition. Nat. Methods.

[59] Wu Y.W., Simmons B.A., Singer S.W. (2016). MaxBin 2.0: An automated binning algorithm to recover genomes from multiple metagenomic datasets. Bioinformatics.

[60] Sieber C.M.K., Probst A.J., Sharrar A., Thomas B.C., Hess M., Tringe S.G., Banfield J.F. (2018). Recovery of genomes from metagenomes via a dereplication, aggregation and scoring strategy. Nat. Microbiol..

[61] Chklovski A., Parks D.H., Woodcroft B.J., Tyson G.W. (2023). CheckM2: A rapid, scalable and accurate tool for assessing microbial genome quality using machine learning. Nat. Methods.

[62] Chaumeil P.A., Mussig A.J., Hugenholtz P., Parks D.H. (2022). GTDB-Tk v2: Memory friendly classification with the genome taxonomy database. Bioinformatics.

[63] Brettin T., Davis J.J., Disz T., Edwards R.A., Gerdes S., Olsen G.J., Olson R., Overbeek R., Parrello B., Pusch G.D. (2015). RASTtk: A modular and extensible implementation of the RAST algorithm for building custom annotation pipelines and annotating batches of genomes. Sci. Rep..

[64] Almagro Armenteros J.J., Tsirigos K.D., Sønderby C.K., Petersen T.N., Winther O., Brunak S., von Heijne G., Nielsen H. (2019). SignalP 5.0 improves signal peptide predictions using deep neural networks. Nat. Biotechnol..

[65] Krogh A., Larsson B., von Heijne G., Sonnhammer E.L. (2001). Predicting transmembrane protein topology with a hidden Markov model: Application to complete genomes. J. Mol. Biol..

[66] Guindon S., Dufayard J.F., Lefort V., Anisimova M., Hordijk W., Gascuel O. (2010). New algorithms and methods to estimate maximum-likelihood phylogenies: Assessing the performance of PhyML 3.0. Syst. Biol..

[67] Anisimova M., Gil M., Dufayard J.F., Dessimoz C., Gascuel O. (2011). Survey of branch support methods demonstrates accuracy, power, and robustness of fast likelihood-based approximation schemes. Syst. Biol..

[68] Hallbeck L., Pedersen K. (1991). Autotrophic and mixotrophic growth of *Gallionella ferruginea*. J. General. Microbiol..

[69] Hoover R.L., Küsel K., Chan C.S. (2025). An organotrophic *Sideroxydans* reveals potential iron oxidation marker genes. Appl. Environ. Microbiol..

[70] Cooper R.E., Finck J., Chan C., Küsel K. (2023). Mixotrophy broadens the ecological niche range of the iron oxidizer *Sideroxydans* sp. CL21 isolated from an iron-rich peatland. FEMS Microbiol. Ecol..

